# A functional magnetic resonance imaging examination of audiovisual observation of a point-light string quartet using intersubject correlation and physical feature analysis

**DOI:** 10.3389/fnins.2022.921489

**Published:** 2022-09-06

**Authors:** Amanda Lillywhite, Dewy Nijhof, Donald Glowinski, Bruno L. Giordano, Antonio Camurri, Ian Cross, Frank E. Pollick

**Affiliations:** ^1^School of Psychology & Neuroscience, University of Glasgow, Glasgow, United Kingdom; ^2^Department of Psychology, University of Bath, Bath, United Kingdom; ^3^Institute of Health & Wellbeing, University of Glasgow, Glasgow, United Kingdom; ^4^La Source School of Nursing, Institut et Haute Ecole de la Santé La Source (HES-SO), Lausanne, Switzerland; ^5^Swiss Center for Affective Sciences, University of Geneva, Geneva, Switzerland; ^6^Institut de Neurosciences de la Timone, UMR 7289, CNRS, Aix-Marseille University, Marseille, France; ^7^Casa Paganini-InfoMus, DIBRIS, University of Genoa, Genoa, Italy; ^8^Centre for Music and Science, Faculty of Music, School of Arts and Humanities, University of Cambridge, Cambridge, United Kingdom

**Keywords:** music, biological motion, fMRI, intersubject correlation (ISC), string quartet

## Abstract

We use functional Magnetic Resonance Imaging (fMRI) to explore synchronized neural responses between observers of audiovisual presentation of a string quartet performance during free viewing. Audio presentation was accompanied by visual presentation of the string quartet as stick figures observed from a static viewpoint. Brain data from 18 musical novices were obtained during audiovisual presentation of a 116 s performance of the allegro of String Quartet, No. 14 in D minor by Schubert played by the ‘Quartetto di Cremona.’ These data were analyzed using intersubject correlation (ISC). Results showed extensive ISC in auditory and visual areas as well as parietal cortex, frontal cortex and subcortical areas including the medial geniculate and basal ganglia (putamen). These results from a single fixed viewpoint of multiple musicians are greater than previous reports of ISC from unstructured group activity but are broadly consistent with related research that used ISC to explore listening to music or watching solo dance. A feature analysis examining the relationship between brain activity and physical features of the auditory and visual signals yielded findings of a large proportion of activity related to auditory and visual processing, particularly in the superior temporal gyrus (STG) as well as midbrain areas. Motor areas were also involved, potentially as a result of watching motion from the stick figure display of musicians in the string quartet. These results reveal involvement of areas such as the putamen in processing complex musical performance and highlight the potential of using brief naturalistic stimuli to localize distinct brain areas and elucidate potential mechanisms underlying multisensory integration.

## Introduction

Observing coordinated action is a complicated task that typically involves processing the relationship between what is seen and what is heard as well as the social relationship between the people being observed. Moreover, it requires this perceptual and social processing to keep up with the ongoing activity. While much is known about the constituent neural mechanisms that might subserve these processes (Grosbras et al., 2012; de Gelder and Solanas, 2021; Schurz et al., 2021), less is known about how the brain responds to naturalistic stimuli that depict coordinated activity of a group of individuals. In this research we use a string quartet as an example of coordinated human activity and examine, with a data-driven exploratory functional Magnetic Resonance Imaging (fMRI) study, the response of musical novices. Besides its relevance to the study of music neuroscience, a musical activity was chosen since ensemble music is widespread and is one of the most closely synchronized human activities and thus provides a relevant and well-defined activity. Musical novice observers were chosen since some studies suggest that experts may employ mechanisms for integrating the sights and sounds of musical activities that are not typical of the general population (Petrini et al., 2011).

Vision has been shown to play an important role in the processing of musical performance, with a meta-analysis of 15 studies of audiovisual music perception revealing a clear influence of vision (Platz and Kopiez, 2012). Ratings such as expressiveness (Wanderley et al., 2005; Dahl and Friberg, 2007; Vines et al., 2011), liking or overall quality of performance differed between audio-only and audiovisual presentation. This finding is consistent with claims that vision will interact with the processing of sound at multiple levels of music processing (Schutz, 2008). One example of an interaction between vision and sound production is provided by evidence that musicians playing solo or in ensemble move differently depending on visibility of the other players (Keller and Appel, 2010; Palmer, 2012). A possible reason for these differences in movement arise from the way that different sensory information interacts with basic timing mechanisms that facilitate coordinated play (Wing et al., 2014). Ensemble performance is thought to arise from entrainment of the musicians (Phillips-Silver and Keller, 2012) to a rhythmic signal (Schachner et al., 2009). Another visual aspect of ensemble playing is the social communication among players afforded by gesture (Glowinski, 2013; Glowinski et al., 2014) that can signal aspects of control, approval and other information. Interaction between biological motion and audiovisual features has been demonstrated in reaction times for detecting visual biological motion, where visual biological motion embedded in noise was detected faster if there was congruent auditory motion (Brooks et al., 2007). Studying this phenomenon in naturalistic stimuli, namely tap dancing, Arrighi et al. (2009) showed that auditory and visual signals integrate in the perception of biological motion only if visual and auditory sequences are synchronized. Moreover, modulation of biological motion perception by kinematic sonification has been shown in gross motor cyclic movements, which were perceived as faster or slower depending on hearing higher or lower global pitch, respectively (Effenberg and Schmitz, 2018). Regardless of the origin of movement and sound differences in the production of ensemble playing, observers are presented with a multisensory signal that is shaped by constraints of ensemble play. In the current research we investigate how a novice group of observers process an ensemble performance by examining brain activity as revealed by fMRI while participants watch and listen to a carefully controlled video of a string quartet ensemble performance (Glowinski, 2013). Brain structures have been shown to entrain to the beat and meter of a musical signal (Iversen et al., 2009; Nozaradan et al., 2011; Fujioka et al., 2012), and the current study informs our understanding of how the audiovisual signal produced by ensemble performance is processed.

Many different auditory features exist in musical stimuli, some of which can be actively distinguished, such as pitch or rhythm and some of which often cannot, such as harmonics. Whilst the term auditory features embodies a multitude of concepts at various levels whose combined effect is larger than its individual components, for the purpose of this study we refer to auditory features as preconceived characteristics of the auditory signal. Although neural correlates of auditory features have often been studied using neuroimaging, neural processing of a continuous stream of auditory features is relatively under examined. Loudness is an often-investigated feature, measured in dB of sound pressure level (SPL), although cortical activations seem to relate to perceived levels of loudness as opposed to the physical sound intensity (Langers et al., 2007; Röhl and Uppenkamp, 2012). Perceptual levels of loudness are therefore measured in sones. For loudness, activation is usually found in the primary auditory cortex (Röhl et al., 2011). Other commonly investigated auditory features include pitch and spatial processing. A meta-analysis by Alho et al. (2014) found activation in the superior temporal gyrus (STG) across 115 fMRI studies that attempted to localize the functions of pitch and spatial location within the auditory cortex. Giordano et al. (2013) found that large portions of the bilateral temporal cortex were related to low level feature processing (pitch, spectral centroid, harmonicity, and loudness). This included auditory areas such as Heschl’s gyrus, the planum temporale (PT) and the anterior STG. In terms of processing, Chapin et al. (2010) found that several areas of cortical activity were correlated with tempo in expressive performances.

A large body of neuroimaging work has correlated different low-level auditory features to specific cortical and subcortical activation using a classic General Linear Model (GLM) design with simple, short stimuli. These studies are able to provide valuable information about the basic mechanisms that underlie these processes, as much of the interference from other factors are excluded to provide more precise and reliable results. However, at the same time the act of excluding this interference limits the extent of knowledge they can provide on natural perceptual processes, whether they are auditory, visual or multimodal. In typical everyday life, scenes are often more complex and, with the inclusion of many objects in a scene as well as the presence of more than one sensory modality, require multisensory integration. This drives research into the use of more complex and naturalistic stimuli.

A considerable amount of research investigating perception with more complex extended stimuli has emerged. However, the use of these complex extended stimuli can be problematic with typical GLM analyses, as GLM analyses typically place constraints on the kind of stimuli that can be analyzed. A significant challenge to studying brain mechanisms of audiovisual perception of a string quartet is how to design the acquisition and analysis of brain data that preserves the intrinsic properties of the string quartet performance. Our solution to this was to use the technique of Intersubject Correlation (ISC) which enables examination of brain activity to complex stimuli extended over minutes (Hasson et al., 2004). ISC is a model-free approach that measures how, among a set of observers, activity at different regions of the brain is correlated among the observers. Evidence has emerged showing the role of correlated activity amongst observers, on a neural, behavioral and physiological level, in shared experience of a musical performance (e.g., Ardizzi et al., 2020; Hou et al., 2020). In this view, ISC could be interpreted as an index of coordinated audience response. This ISC can be extensive as demonstrated by the original study of Hasson et al. (2004) which showed that when a group of observers watched an excerpt of Sergio Leone’s film *The Good, the Bad and the Ugly* (1966), ISC was found in about 45% of cortex. One advantage of ISC is that it is independent of the level of activity within the brain, which circumvents the issue that the hemodynamic response function varies not only between brain areas, but also between subjects (Jääskeläinen et al., 2008). It has also been shown across a variety of studies to be a reliable method to investigate neural activity during complex stimuli, when compared to classic modeling approaches using the GLM (Pajula et al., 2012). ISC has been used with fMRI data to study a variety of topics including memory (Hasson et al., 2008a), perception of film (Hasson et al., 2008b), temporal receptive windows (Hasson et al., 2008c) and communication (Hasson et al., 2012; Rowland et al., 2018). It has also been applied to the study of group differences due to development (Cantlon and Li, 2013), autism (Hasson et al., 2009) and the expertise of CCTV operators in judging harmful intent (Petrini et al., 2014). Finally, the ISC approach has also been adapted for use in electroencephalogram (EEG) (Dmochowski et al., 2012) and functional near infrared spectroscopy (fNIRS, Da Silva Ferreira Barreto et al., 2020) and applied to predicting audience preferences of different television broadcasts (Dmochowski et al., 2014) and tracking information propagation as a function of attention while listening to and reading stories (Regev et al., 2019).

Audiovisual musical performance has not been widely studied using ISC. One study investigated shared physiological responses across audience members during a live string quintet performance and explored ISC activity using musical features (Czepiel et al., 2021). Moreover, two studies yielding somewhat divergent results relate to what one might expect to find. One study with novice western observers used ISC to compare activity during audiovisual presentation of a solo dance to the conditions of audio (music) and video (dance) presentation (Jola et al., 2013). This study used novice western observers and Indian dance; a Bharatanatyam dancer accompanied by the music of Theeradha Vilaiyattu Pillai by Subramania Bharathiyar. Results showed that compared to unisensory conditions, audiovisual presentation of dance and music showed greater areas of ISC in bilateral visual and auditory cortices, with activity extending into multisensory regions in the superior temporal cortex. Regions of ISC were not found outside of occipital and temporal cortex. Another study (Abrams et al., 2013) with novice western observers used ISC to examine activity during listening to symphonic music of the late baroque period. Results showed that listening to music activated an extensive network of regions in bilateral auditory midbrain and thalamus, primary auditory and auditory association cortex and right lateralized structures in frontal and parietal cortex and motor regions of the brain. The difference in ISC found to music stimuli is striking between the two studies. While the difference in area of ISC can potentially be explained by differences in auditory properties of the music or familiarity of the participants to the genre of the music, or even particular aspects of how maps of ISC were obtained statistically, it raises the question of how much the area of ISC might vary between different musical pieces. Another question that arises is whether the ISC found in visual cortex with dance will be found with ensemble music performance. Achieving regions of ISC has been argued to rely upon the different viewers observing the same thing at the same time (Hasson et al., 2004). In carefully edited Hollywood-style movies (Hasson et al., 2008b) it is possibly not surprising that viewers are watching the flow of events in synchrony. For dance (Jola et al., 2013) it was also argued that choreography typically directs observers’ viewing. These results are distinctly different from reports of ISC based on eight participants watching an unedited video of an outdoor concert from a fixed viewpoint, where less than 5% of cortex (primarily in auditory and visual cortices) was found in the ISC maps (Hasson et al., 2010). For the case of observing a string quartet, it is not clear whether the activity of the ensemble will produce brain activity in a way that would enable regions of ISC to be found extensively in visual, or other brain regions.

In this study we use fMRI to examine the brain response to hearing and seeing an ensemble performance of a string quartet. Visual presentation of the performance is achieved through display of the musicians as stick figures derived from motion capture data. Stick figures were chosen over natural video since the stick figures allow us to control the visual salience of the players so there would not be a preference to view any particular player based on surface appearance. While being scanned participants had no task and were told simply to enjoy the performance. The data were analyzed using ISC to reveal which brain areas had correlated activity among the group of observers. They were also examined to see whether a relationship could be found between brain activity and physical measures of the sound and vision of the string quartet. Brain regions revealing ISC amongst observers indicate how brain activity becomes time-locked to external stimuli (Hasson et al., 2004), with sensory regions typically showing activity synchronized to audio (Abrams et al., 2013) and visual features (Noble et al., 2014). Examining correlation of brain activity with audio and visual signals enables us to more closely examine the influence of different features. Of particular interest is whether ISC maps would be found in visual processing areas since experiencing the string quartet by free viewing does not require a group of observers to share a common visual processing strategy. In addition, this study aims to use an audiovisual musical stimulus to investigate the underlying neural basis of a variety of auditory features. Similar to the experiment of Abrams et al. (2013), this study will use an extended piece of music to investigate these features. However, this study will also introduce vision using an upper body stick figure display of the musicians as they play their instruments. As in Alluri et al. (2012) an inter-subject correlation will be done, to investigate the ISC map resulting from the stimulus. Examination of the correlation of auditory and visual features to brain activity will also be conducted to investigate how much of the ISC can be explained by the different features. The auditory feature analysis will consist of an analysis of three different time-varying auditory features: loudness, spectral centroid (Spectral Center of Gravity or SCG) and sound periodicity (Ap0). Loudness is primarily a measure of the psychological correlate of the amplitude of the sound wave. SCG describes the average amplitude of a sound and is connected to the brightness, or timbre of a sound. Sound periodicity is a measure of harmonics. The features investigated in the current study are based on features extracted by Giordano et al. (2013). They measured SCG, loudness and Ap0 in short naturalistic stimuli, although they refer to Ap0 as harmonicity (HNR). Visual feature analysis will consist of an analysis of speed and movement similarity, where speed is a group measure of the magnitude of the motion of the different players in the ensemble and movement similarity is the similarity of the motion of the different players.

## Materials and methods

### Participants

The fMRI data were collected from 18 healthy participants (eight males) with a mean age of 26 years. All subjects had normal hearing and normal or corrected to normal vision. All participants were right-handed, as assessed by the Edinburgh handedness inventory (Oldfield, 1971). Recruitment information included criteria for non-expert or novice musical listeners. Participants either received a number of course credits, or a small cash payment for their participation. Ethical approval for this study was obtained from the College of Science and Engineering ethics committee, at the University of Glasgow.

### Stimulus

**FIGURE 1:**
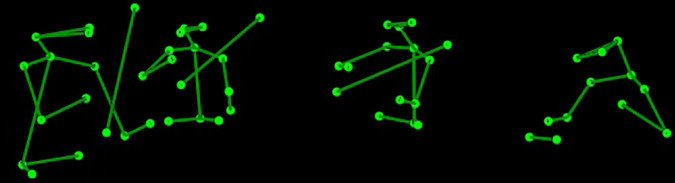
The upper body stick figure display of the string quartet the ‘Quartetto di Cremona’.

The stimulus was a 116 s audiovisual presentation of a string quartet; musicians were displayed as stick figures of the upper body and bow (Figure 1) in a movie presented at 25 fps. The size of the stimulus was approximately 22.8 degrees of visual angle wide and 7.4 degrees high. The width of a player was approximately 3.7 visual degrees and the distance between players was 4.6, 6.5, and 6.5 degrees going left to right, respectively, from the first violin. The visual stimulus was created from 3D motion capture of a recording of the string quartet the ‘Quartetto di Cremona,’ playing the allegro of String Quartet, No. 14 in D minor by Schubert. The particular piece was selected because it exemplifies a range of interaction within the quartet, including where musicians tend to play in unison, but over which the first violinist emerges progressively through a subtle original motif. Another section used a fugato style, where there is no leading part as such. Moreover, the piece is a staple of the quartet’s repertoire and thus could be played consistently at a high quality. The recording has been used in previous research of Glowinski (2013) and Glowinski et al. (2014) and is described in more detail by Badino et al. (2014). Recording was done by the InfoMus research group at the University of Genoa and took place in a 250-seat auditorium that provided an ecologically valid location for the performance. Audio and visual information were synchronized using EyesWeb XMI software (Camurri et al., 2005; Glowinski, 2013).

### Auditory analysis of the string quartet

Auditory analysis was performed using custom-made Matlab scripts. Three different features were extracted from the audio of the stimulus, for the feature analysis: Loudness (measured in sones), SCG (measured in Equivalent Rectangular Bandwidth rate units) and Ap0 (measured in dB). Loudness and SCG were derived from time-varying specific loudness of the sound signals that were computed using the model of Glasberg and Moore (2002). Ap0 was computed using the YIN model of De Cheveigné and Kawahara (2002).

For each feature the median statistics were obtained for each 2 s segment of the 116 s performance. This provided 58 values for each feature that corresponded to the number of functional brain volumes obtained. However, only the first 57 values were used due to concerns of accumulated timing errors leading to a mismatch in the synchronization between the scanner volumes and the movie file that could lead to artifactual computations in the final volume. Analyses were then conducted for each of the features (Ap0, loudness and SCG) to see whether their time varying activity could account for changes in the fMRI signal. Exploratory analyses revealed little difference between the mean and the median as well as the standard deviation and interquartile range. Therefore, following (Giordano et al., 2013) we present only results based on the median.

### Visual analysis of the string quartet

Visual analysis was performed using custom-made scripts in Matlab. A visual analysis of the kinematic features of the string quartet focused on analysis of the speed of the different player’s motions as well as the similarity of the motion of the different players. Given the complex motion of each player there are many possibilities of what motion feature to examine. We chose to focus on the three-dimensional motion of the bow of each player to characterize movement. The bow is the end-effector of a complex kinematic chain and has been useful to characterize emotional arm movements (Pollick et al., 2001) as well as musical conducting movements (Luck and Sloboda, 2009; Wöllner and Deconinck, 2013). To perform this, we took the original 100 Hz data sample and first calculated the average *x*–*y*–*z* position of the two markers on the bow for each player. We then filtered these data with a 6th order dual-pass Butterworth filter with a cutoff frequency of 10 Hz to remove high frequency noise from the data. These filtered data were then differentiated to find the three-dimensional velocity vector. From this velocity vector, we calculated the scalar value of speed, corresponding to the rate at which a bow was moving through three-dimensional space. This calculation of speed was done for each bow. Because the sampling rate for the fMRI was one sample every 2 s, we averaged the speed over 2 s epochs to find a total of 58 average speed values to represent a visual speed signal that could be compared to brain activity. We were also interested in whether the similarity of the four players might be related to brain activity, and to examine this we performed a measure of movement similarity based on a calculation of the average correlation of speed between players for each of the 58 epochs calculated. As there were four musicians there were six pairwise comparisons to make between players. Each of the six comparisons were calculated and the median was chosen as an overall measure of movement similarity. However, as with the auditory analysis, only the first 57 values were used due to concerns of accumulated timing errors leading to a mismatch in the synchronization between the scanner volumes and the movie file that could lead to artifactual computations in the final volume.

### Procedure

The string quartet stimulus was presented in a single run using the software Presentation (Neurobehavioral systems, Inc.). The video of the string quartet performance was preceded and followed by 20 s of a blank screen, resulting in a run consisting of 20 s of blank screen, followed by 116 s of stimuli and ending on 20 s of blank screen. The scanning session included acquisition of fMRI data for other experiments not related to this study and this experiment came second in the run order after the first experiment of approximately 5 min. At the completion of all functional data experimental runs an anatomical scan was performed. The video was projected using an LCD projector on a translucent screen, while participants watched them in an angled mirror in the scanner. The soundtrack of the video was presented using in-ear headphones (model S14 by Sensimetrics, Malden, United States). Participants were given no explicit task when experiencing the videos, they were instructed simply to relax and enjoy the video. After scanning, participants were asked about their subjective experience of the performance and none reported familiarity with the piece.

### Data acquisition and preprocessing

Data were acquired using a Siemens 3 Tesla TIM Trio magnetic resonance imaging scanner (Erlangen, Germany). Functional scans were taken to obtain brain volumes with 32 sagittal slices covering the brain volume, with 3 mm × 3 mm × 3 mm voxel size (Echo Planar 2D imaging PACE; TR = 2,000 ms; TE = 30 ms; imaging matrix of 70 × 70). The 116 s stimulus was contained in 58 volumes and was preceded and followed by 10 volumes for a total of 78 volumes. At the end of the functional scans, high resolution anatomical T1 weighted 3D magnetization prepared rapid acquisition gradient echo (MP- RAGE) images were acquired, with 192 sagittal slices, a 256 × 265 matrix size and a 1 mm × 1 mm × 1mm voxel size and a TR of 1,900 ms.

The data files were pre-processed using Brainvoyager QX V2.8 (Brain Innovation B.V., Maastricht, Netherlands). The functional images underwent slice scan time correction, using cubic spline interpolation. 3D Motion correction was then also applied using trilinear detection and sinc interpolation. This was followed by normalization of functional scans into the common Talairach space (Talairach and Tournoux, 1988), and co-registration of functional and anatomical data. Spatial smoothing was then obtained using a Gaussian filter with a full width and half maximum (FWHM) of 6.00 mm. Finally, the functional data were trimmed to contain only the 58 volumes during the string quartet performance and these data were converted into MATLAB (The Mathworks, Natick, MA, United States) based mat files for ISC analysis.

### Inter-subject correlation analysis

The ISC analysis was conducted using MATLAB and the techniques developed by Kauppi et al. (2010, 2014) and Pajula et al. (2012). As in Kauppi et al. (2010), an ISC test statistic was derived by computing Pearson’s correlation coefficient voxel-wise across the time-courses of every possible subject pair and then averaging the result across all subject pairs. A non-parametric bootstrap test with 1 million resamples was conducted to test against the null hypothesis that the test statistic was the same for unstructured data. A *q*-value threshold of 0.05 was used to correct for multiple comparisons using False Discovery Rate (FDR). A further cluster threshold of 108 mm^3^ (four functional voxels) was applied. All 58 volumes were used in the calculation of ISC.

### Relating auditory and visual features to brain activity

We used the following procedure to test whether the visual features of speed and movement similarity as well as the auditory features (Ap0, loudness, SCG) related to changes in the BOLD signal. Z- transformed data from each feature were convolved with the modeled hemodynamic response function (HRF). This gave a predictor for brain activity for each of the 57 time points considered. This model was then applied to the entire brain. Activation for the statistics of each of the features was analyzed using a random effects GLM, with Z- transform. The activations reported arise from use of an FDR adjusted criterion threshold of q(FDR) < 0.01.

## Results

### Intersubject correlation analysis

**FIGURE 2:**
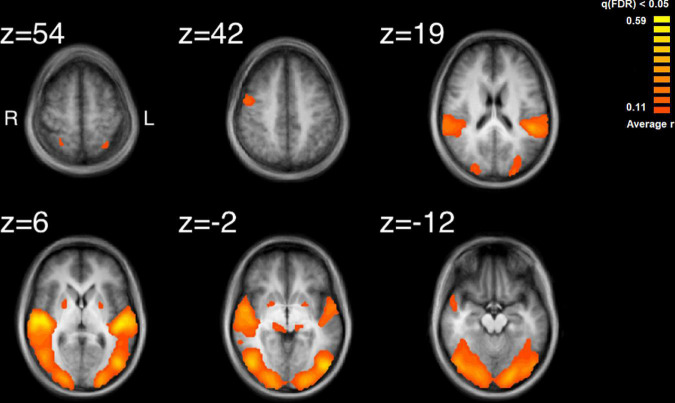
Results of the inter-subject correlation obtained from the average of all possible pairwise correlation, r, values. Significant regions of ISC include the bilateral superior temporal gyrus, peaking in the right STG, bilateral, occipital areas, the right precentral gyrus and regions in the midbrain. Images are shown in radiological format: left brain to image right and right brain to the image left.

The results of the ISC analysis are shown in Figure 2 and details of the cluster and peak voxel for this ISC map are provided in Table 1, along with information about the location of the peak voxel by the Talairach Daemon (Lancaster et al., 1997, 2000). A total of 8 ISC clusters were found across the 18 participants. These occurred primarily in auditory and visual sensory areas. These clusters contained a total of 154,728 voxels, or 9.9% of the total 1,562,139 anatomic voxels (1 mm^3^) included in the entire brain volume.

**TABLE 1:** Results of the inter-subject correlation (ISC) analysis for watching and listening to the point-light string quartet.

Structure	Hemisphere	Talairach coordinate (*x, y, z*)	Number of voxels	Peak statistic	BA
Superior temporal gyrus	Right	(47, −14, 3)	149747	0.59	22
Precentral gyrus (PG)	Right	(50, −5, 46)	1508	0.20	4
Precuneus	Right	(27, −49, 51)	124	0.11	7
Globus pallidus	Right	(20, 0, −3)	749	0.13	
Medial geniculate	Right	(14, −26, −3)	955	0.17	
Nucleus (MGN)					
Medial geniculate	Left	(−13, −26, −3)	424	0.16	
Nucleus (MGN)					
Putamen	Left	(−22, 1, 0)	558	0.12	
Superior parietal lobule (SPL)	Left	(−31, −53, 48)	663	0.13	7

The number of voxels is the total voxel (1 mm3) count. The x, y, z coordinates are of the peak voxel in Talairach space. The peak statistic is that value in the cluster which, for each voxel, had the greatest value of average correlation. BA refers to Brodmann’s Area.

The largest cluster had a peak in the right STG and extended bilaterally through regions in the occipital cortices, to the left STG. This cluster thus contained extensive regions of brain areas known to be involved in audio, visual and audiovisual processing. More focal clusters were also found in cortical and subcortical structures. In the cortex, clusters were found both in motor cortex (BA4) at the boundary of Brodmann Area 6 and bilateral parietal cortex. Subcortical clusters were found bilaterally both in thalamus and the basal ganglia. In the thalamus activity was found in an auditory region, the medial geniculate nucleus (MGN). In the basal ganglia activity was found in the right hemisphere to have peak ISC in the globus pallidus and extended into the putamen; in the left hemisphere it had peak ISC in the putamen and appeared contained in this structure.

### Auditory feature analysis

**FIGURE 3:**
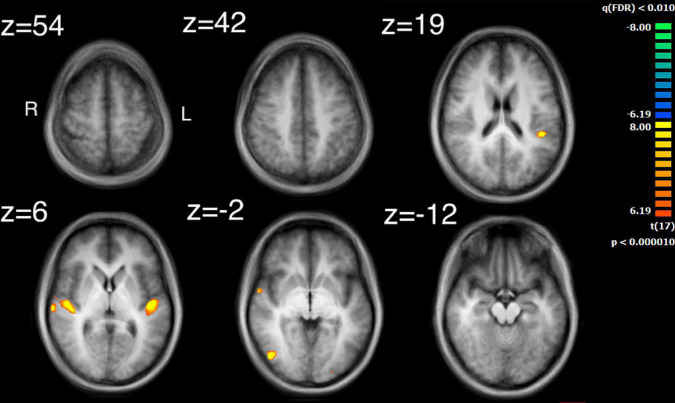
Activity for the median values of SCG. Significant regions of activity include the left and right superior temporal gyrus, left inferior occipital gyrus and right inferior temporal gyrus.

**FIGURE 4:**
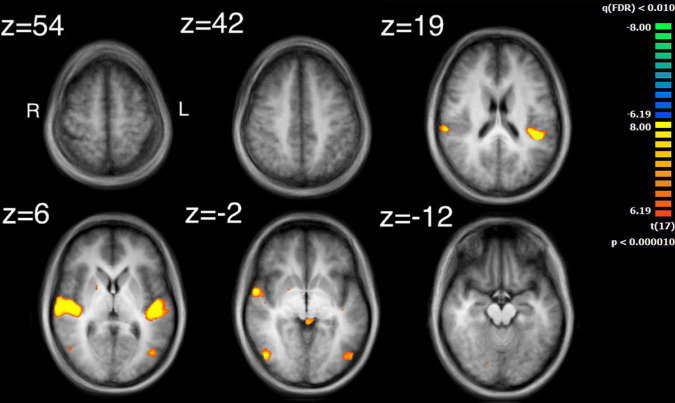
Activation for the median values of loudness. Significant regions of activity include the bilateral superior temporal gyrus, the right inferior temporal gyrus and left middle occipital gyrus.

The auditory analysis investigated the median of the values of the three features, Ap0, SCG, and loudness (the trace of these features as a function of time can be seen in Supplementary Figure 1). The results for the median values of Ap0 are not reported, as no related neural activity was found for this feature. Both the features of SCG and loudness were found to predict BOLD activity. A total of 8 significant clusters were found for the median values of SCG, across both hemispheres (Figure 3 and Table 2) with a total of 24,499 voxels. The most widespread activation came from the right STG with a total voxel count of 8,004. Extensive activation was also found in the left STG, with the second highest voxel count. The right inferior temporal gyrus, cerebellum and the left inferior occipital gyrus also showed marked activations. Several large areas of activation were found for the convolution of the HRF with loudness (Figure 4 and Table 3) with a total voxel count of 51,297. The largest two clusters occurred bilaterally in the STG (Brodmann areas 13, 41). The right inferior temporal gyrus along with the lentiform nucleus also contained large clusters with high voxel counts.

**TABLE 2:** Results of the auditory feature analysis for the median values of SCG.

	Structure	Hemisphere	Talairach coordinate (*x, y, z*)	Number of voxels	Peak statistic	BA
**SCG**						
	Superior temporal gyrus	Right	(51, −13, 1)	8004	9.601	22
	Inferior temporal gyrus	Right	(42, −70, 1)	3156	10.813	19
	Tuber, cerebellum	Right	(49, −58, −23)	113	5.926	
	Superior frontal gyrus	Right	(21, 59, 32)	314	−6.620	9
	Declive, cerebellum	Right	(15, −76, −14)	3048	7.420	
	Inferior occipital gyrus	Left	(−24, −88, −2)	1301	6.783	18
	Superior temporal gyrus	Left	(−42, −34, 16)	7476	10.396	41
	Middle occipital gyrus	Left	(−39, −67, 4)	1087	6.789	37

The number of voxels is the total voxel (1 mm^3^) count. The *x*, *y*, *z* coordinates are of the peak voxel in Talairach space. The peak statistic is that value in the cluster which, for each voxel, had the greatest value of average correlation. BA refers to Brodmann’s Area.

**TABLE 3:** Results of the auditory feature analysis for the median values of loudness.

	Structure	Hemisphere	Talairach coordinate (*x, y, z*)	Number of voxels	Peak statistic	BA
**Loudness**						
	Superior temporal gyrus	Right	(45, −19, 7)	15982	11.554	13
	Precentral gyrus	Right	(51, −1, 43)	472	5.433	6
	Inferior temporal gyrus	Right	(46, −70, −2)	4184	8.633	37
	Tuber, cerebellum	Right	(49, −58, −23)	921	6.809	
	Declive, cerebellum	Right	(24, −58, −17)	4418	6.896	
	Lentiform nucleus – lateral globus pallidus	Right	(18, 2, 1)	3805	7.502	
	Lingual gyrus	Right	(15, −88, 4)	144	4.812	17
	Culmen, cerebellum	Left	(−6, −31, −5)	1460	7.709	
	Medial frontal gyrus	Left	(0, −4, 64)	517	5.570	6
	Declive, cerebellum	Left	(−5, −85, −20)	238	5.208	
	Lentiform nucleus – putamen	Left	(−21, 2, 10)	1090	6.784	
	Superior temporal gyrus	Left	(−42, −34, 16)	14607	14.868	41
	Middle occipital gyrus	Left	(−42, −67, 4)	3306	7.854	37
	Tuber	Left	(−51, −52, −23)	153	5.143	

The number of voxels is the total voxel (1 mm^3^) count. The *x*, *y*, *z* coordinates are of the peak voxel in Talairach space. The peak statistic is that value in the cluster which, for each voxel, had the greatest value of average correlation. BA refers to Brodmann’s Area.

### Visual feature analysis

**FIGURE 5:**
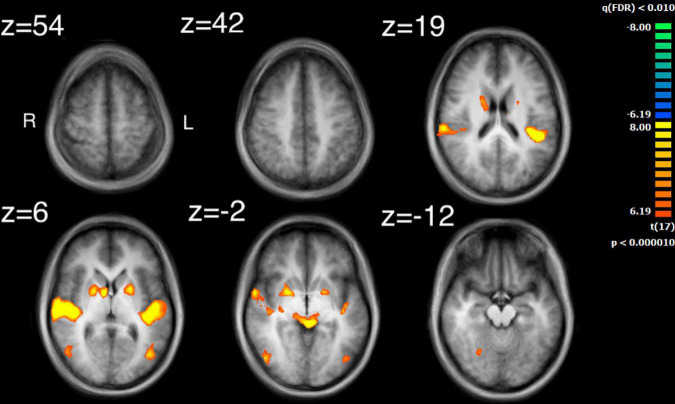
Activation for the median values of speed. Significant regions of activity include the bilateral posterior insula extending into superior temporal gyrus as well as right inferior temporal and left middle occipital gyrus in addition to numerous subcortical structures.

**FIGURE 6:**
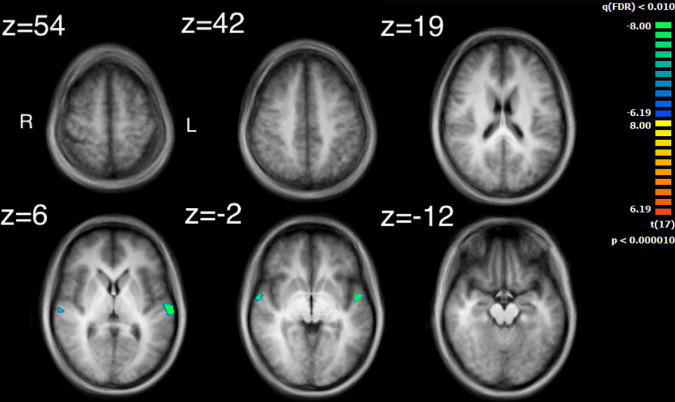
Activation for the median values of movement similarity. Regions of activity are in the left and right superior temporal gyrus. Note that clusters found for movement similarity are negatively correlated.

The visual analysis investigated the median of the values of the two features, bow speed and a measure of the movement similarity of the players (the trace of these features as a function of time can be seen in Supplementary Figure 1). For bow speed, results showed that the median values of bow speed predicted large bilateral clusters of activation in subcortical structures as well as bilateral activation in the posterior insula that extended into the STG (Figure 5 and Table 4). The larger of the two clusters was found in the left hemisphere and the smaller was found in the right hemisphere. For the movement similarity analysis, four clusters of activation were found bilaterally, contained within the STG (Figure 6 and Table 5). However, in distinction to the other features examined, movement similarity clusters were negative, meaning that BOLD activity was inversely related to movement similarity; decreasing movement similarity predicted increases in BOLD activity and vice versa. The largest clusters were found in the right hemisphere, with smaller clusters found in the left hemisphere (Brodmann 22).

**TABLE 4:** Results of the visual feature analysis for the median values of speed.

	Structure	Hemisphere	Talairach coordinate (*x, y, z*)	Number of voxels	Peak statistic	BA
**Speed**						
	Insula	Right	(39, −19, 4)	11601	13.064	13
	Inferior temporal gyrus	Right	(46, −70, 1)	1370	8.147	19
	Declive	Right	(27, −61, −17)	720	7.471	
	Lentiform nucleus – lateral globus pallidus	Right	(18, 2, 1)	4577	9.927	
	Thalamus – pulvinar	Left	(−3, −31, −2)	2416	10.947	
	Superior frontal gyrus	Right	(3, −4, 64)	514	7.174	6
	Lentiform nucleus – putamen	Left	(−21, 2, 10)	2335	10.842	
	Insula	Left	(−42, −37, 19)	10073	23.486	13
	Middle occipital gyrus	Left	(−42, −67, 4)	1242	8.390	37

The number of voxels is the total voxel (1 mm^3^) count. The *x*, *y*, *z* coordinates are of the peak voxel in Talairach space. The peak statistic is that value in the cluster which, for each voxel, had the greatest value of average correlation. BA refers to Brodmann’s Area.

**TABLE 5:** Results of the visual feature analysis for the median values of movement similarity.

	Structure	Hemisphere	Talairach coordinate (*x, y, z*)	Number of voxels	Peak statistic	BA
**Movement similarity**						
	Superior temporal gyrus	Right	(60, −19, 4)	178	−8.227	22
	Superior temporal gyrus	Right	(57, −4, 1)	173	−8.855	22
	Superior temporal gyrus	Left	(−51, −4, 1)	441	−10.211	22
	Superior temporal gyrus	Left	(−63, −19, 4)	665	−12.008	22

The number of voxels is the total voxel (1 mm^3^) count. The *x*, *y*, *z* coordinates are of the peak voxel in Talairach space. The peak statistic is that value in the cluster which, for each voxel, had the greatest value of average correlation. BA refers to Brodmann’s Area.

### Comparing intersubject correlation to auditory and visual feature analyses

Comparison of the ISC analysis (Figure 2 and Table 1) and the feature analyses (Figures 3–6 and Tables 2–6), show that the activation where ISC is significant encompasses several regions of activation found in the feature analyses. The STG was one of the most extensive regions of activation found in the ISC. STG clusters were found in the analysis of the median values of loudness, SCG and movement similarity. Activation found in the midbrain for the ISC only encompassed activation found for the median values of loudness and bow speed. Regions of correlated activity in the lentiform nucleus, specifically in the globus pallidus, for the ISC corresponded to significant regions of activation for the median values of SCG, loudness, and speed, although this activation was stronger for speed than for loudness or SCG.

**TABLE 6:** Pearson correlations between all physical auditory and visual features.

	Ap0	SCG	Loudness	Speed	Movement similarity
Ap0	1				
SCG	0.229	1			
Loudness	–0.027	0.763***	1		
Speed	0.171	0.659***	0.879***	1	
Movement similarity	–0.071	–0.034	0.116	0.191	1

***Indicates *p*-value < 0.001.

### Correlation of physical features

Values for the physical auditory and visual features were correlated (Table 6). Strong correlations were observed between loudness and SCG, loudness and speed, as well as speed and SCG. Movement similarity and Ap0 both showed a lack of correlation to other visual and auditory features, corresponding to the lack of neural results observed for Ap0 but contrary to the observed brain activity in the left and right STG for movement similarity.

## Discussion

Here we showed brain activity, revealed by fMRI, correlated among a group of observers when they experienced the audiovisual presentation of a string quartet musical performance. The present study had two aims. The first aim was to investigate which regions were found in participants’ ISC maps when watching an audiovisual video of a string quartet. Three auditory features (Ap0, SCG, and loudness) and two visual features (speed and movement similarity) were then extracted from the audiovisual stimulus and, subsequently, we examined how much of the ISC could be explained by the regions of activity correlated with these auditory and visual features.

### Intersubject correlation maps

Intersubject correlation was found as expected in both auditory and visual sensory areas and extended into audiovisual regions in superior temporal cortex (Ethofer et al., 2006). Further regions of ISC were found in subcortical, parietal and frontal regions which implicates an extended set of brain areas involved in the processing of complex audiovisual stimuli. Several regions of the ISC map can be attributed to processing the audio of the string quartet. For example, the medial geniculate nucleus is known for auditory processing in the thalamus (Jiang et al., 2013). In addition, there were substantial regions of ISC over bilateral primary and secondary audio areas. Indeed, the highest values of ISC found were located in the right STG (BA22), which is an important area for the processing of basic auditory features. These results are consistent with previous measurements of ISC for listening to music and watching dance with music (Abrams et al., 2013; Jola et al., 2013). Whether this auditory processing revealed by the ISC was enhanced by the visual signal is a possibility, as EEG evidence has shown that visual input can facilitate audio processing (Klucharev et al., 2003; Van Wassenhove et al., 2005; Jessen and Kotz, 2011). Similarly, Jola et al. (2013) reported more extensive ISC in auditory regions during audiovisual presentation of dance with music than with music alone. The bilateral ISC found in the parietal cortex around the intraparietal sulcus has been reported previously in listening to music (Abrams et al., 2013). Similar to these earlier findings we also report a greater area of ISC in the left hemisphere. An interpretation of this parietal activity is that it reflects tracking the structure of the sound (Levitin and Menon, 2003). However, similar parietal activity has also been implicated in attentional tracking of visual objects (Culham et al., 1998).

Several regions of the ISC map can be attributed to processing the video of the string quartet. Evidence of visual processing comes from extensive ISC found bilaterally from regions of primary visual cortex through to secondary visual areas including those involved in motion processing. These findings are consistent with previous examinations of ISC for visual and audiovisual presentation of dance (Jola et al., 2013; Herbec et al., 2015; Reason et al., 2016; Pollick et al., 2018). In particular, the middle temporal region contained in the ISC map has been found to covary with the global motion of the dancer (Noble et al., 2014). This finding might help to explain how substantial correlation was found throughout the visual cortex during free viewing of the string quartet. One consideration is that with free viewing there is no reason to believe that observers would be viewing the same musician at the same time, which is a basic requirement for visual activity to be correlated across observers. However, one possibility is that due to the large receptive fields reported for the perception of biological motion (Ikeda et al., 2005; Thompson and Baccus, 2012), extending 4 degrees from fixation, that particularly for the more closely spaced players there is overlap and response is being driven by a mixture of signals from multiple players. Similarly, visual neurons responsive to biological motion have been shown to integrate over long time periods (Neri et al., 1998) and thus activity might further be combined across fixations on different players. In contrast, there is evidence from eye-tracking studies that music, and sudden changes in music, can affect visual attention (Mera and Stumpf, 2014), as shown in reading (Zhang et al., 2018). Thus, another possibility is that at key events in the musical composition, musical features directed visual attention toward the same musician, though any effects are likely to be difficult to disentangle, given the documented influence of expertise, familiarity and immediate musical goal on visual processes involved in music reading in performance (Puurtinen, 2018; Vandemoortele et al., 2018; Fink et al., 2019; Bishop et al., 2021). Further studies examining eye movements could possibly help to resolve this issue.

Several regions of the ISC map can be attributed to a role for the motor system in processing the string quartet. Evidence for the involvement of motor systems come from the finding that regions of the motor cortex and basal ganglia were contained in the ISC map. The basal ganglia, including the putamen are important regions that connect to the primary motor cortex, premotor cortex and the supplementary motor area. Although ISC has previously been observed in premotor cortex and supplementary motor area when listening to classical music (Abrams et al., 2013), the finding of ISC in the basal ganglia is novel. One possible reason why ISC in the putamen was not found previously for listening is that visual stimulation is critical. However, presentation of music and dance (Jola et al., 2013) also did not reveal ISC in subcortical regions and thus visual motion coordinated with sound does not seem sufficient. Potentially a key difference between dance and a string quartet is that the dance is choreographed to the music but does not produce the music; in distinction the string quartet produced the sounds and thus there was a clear mapping between the visual and auditory stimuli. If, for example, vision of the string quartet provided a useful signal to predict the beat structure of the music then this could explain the result since the putamen has been shown to modulate its activity with prediction of the beat structure of an auditory signal (Grahn and Rowe, 2009, 2013). Similarly, greater activation has been found in the premotor cortex when listening to preferred versus non-preferred tempos (Kornysheva et al., 2010).

### Auditory and visual feature analysis and their comparison to intersubject correlation maps

Investigation of regions of activity for the auditory and visual features revealed several areas, such as STG, which appeared in the ISC maps. This suggests that some of the ISC could be explained by the auditory features contained within the music or visual features displayed by the string quartet. Median values of both SCG and loudness activated the left STG in the primary auditory cortex, although activation from loudness had a much larger cluster of activation. Median values for movement similarity showed bilateral activation of the STG, with larger clusters in the right hemisphere. ISC in the STG can probably be partly explained by the features of SCG, loudness, and movement similarity. However, the activity found in the STG by the ISC was much more widespread, whereas peak activation for loudness and SCG was centered on the primary auditory cortex. These results support the findings of Giordano et al. (2013) who also found activation for loudness in the primary auditory cortex lateralized to the left hemisphere. Alluri et al. (2012) found more extensive activation in the STG for a variety of features, including activation in area 22 of the STG, bilaterally, for the features: fullness, brightness, activity and timbral complexity. The present study also found that bilateral activation in the STG was inversely related to movement similarity. Taken together, this suggests that other features might additionally explain ISC found in the STG, as well as ISC of the STG in the right hemisphere. The findings also are consistent with the idea of hierarchical processing in auditory perception, with low level features being processed by core auditory areas (Kaas and Hackett, 2000). In addition to the STG’s involvement in auditory processing, it has also been implicated as a critical structure in social cognition. Research by Ceravolo et al. (2021) revealed a widely ramified network of brain regions in decoding or inferring communicative intent in musical context, where STG took a key role even under altered perceptual conditions. Adjacent to the STG is the superior temporal sulcus, which has been implicated in visual processing and social cognition (Allison et al., 2000). Related to this observation is the fact that the brain areas related to the feature of movement similarity were found along the STG in BA22, which comprises the superior temporal sulcus. Suggesting that this movement similarity relates to social processing among the players. Overall, our results confirm and extend, using naturalistic stimuli, how this specific underlying process may also support the perception of musical ensembles.

Significant activity in the midbrain for loudness and speed could explain the ISC map regions found in the right midbrain for the ISC. ISC in the lentiform nucleus, specifically in the globus pallidus, in both the left and right hemispheres could be explained by SCG, loudness, and speed. This is a subcortical structure that seems to play a role in motor processes, although research into this area is limited and has been conducted mostly on primates and rats, with focus on neurotransmitters in nervous disorders such as Catalepsy and Parkinsonism (Wei and Chen, 2009; Galvan et al., 2010).

There was a large overlap of activity between the features SCG, loudness and speed. This may be due to the significantly high correlations found between these features. Some regions found in the ISC maps can be linked to regions of activity found for SCG, loudness, speed and movement similarity. However, other unaccounted for activations may have occurred in response to features that were not investigated in this analysis, such as pitch, or rhythm.

Results of the present study support the findings of previous studies of other complex performances. Bilateral clusters of activation were found in the occipitotemporal area (BA19 and BA37), for median values of loudness, SCG and speed. This area has previously been implicated in human motion perception in previous research relating the visual motion of the silhouette of a dancer to brain activity (Noble et al., 2014). Jola et al. (2013) also found ISC in this area when participants watched the same dance as used by Noble et al. (2014). Grosbras et al. (2012) found that this occipitotemporal area had the greatest chance of being activated in biological motion perception experiments. Given the established role of visual motion in activating this brain region it can be questioned whether the finding of brain activity related to loudness and SCG arises purely from their correlation with visual speed. One possibility would be that the relationship between speed and auditory properties is obligatory due to physical coupling of the features. However, the study of sound production of violins indicates that SCG and speed are positively correlated but that loudness is not correlated to speed (Edgerton et al., 2014). Instead, loudness is predominately modulated by moving bow position toward the body of the instrument. A speculative explanation would be that the coupling of speed is connected to the expressive nature of the movements, which is consistent with the finding that the peak of activity related to speed was found in the posterior insula. Overall, significant open questions remain about whether or not it may be possible to identify overall neural responses to auditory features that correspond to higher-level architectonic musical structures.

## Limitations

The use of the extended audiovisual string quartet stimulus in this study provides valuable information about the effects of an extended multi-modal stimulus on auditory feature analysis. It does, however, make the results of the auditory analysis less clear. The use of extended complex stimuli can be problematic, in that it is difficult to separate out which perceptual or cognitive processes might correspond to which features. It might be that a particular cortical area is found to have a correlation with a perceptual feature, but it is not necessarily the case that these two are causally related. There may well be a third or hidden variable that drives the neural activity. This can be especially unclear when the functions of cortical or subcortical areas are not well understood, or when these areas have been implicated in a variety of different processes.

Given that not all of the vast number of previously studied auditory features were explored in this study, regions of activation shown on the inter-subject correlation that did not correspond to an auditory feature in this study, may well be implicated in other auditory features not investigated here. Many other studies have placed an important emphasis on pitch, for example. These were measured in both Alluri et al. (2012) and Giordano et al. (2013). This study did not allow for a measurement of pitch, due to the nature of the auditory stimulus, a distinct pitch would have very rarely been able to be extracted, leading to confusion over which of the sound’s frequencies would have determined the pitch. Rhythm and tonality were also measured by Alluri et al. (2012), but were not considered in this study.

Expertise can also have a great effect on perception. Previous studies have demonstrated the effect that expertise can have on perception of performances of dance and music. Calvo-Merino et al. (2005) for example showed that experts in one style of dance exhibited greater bilateral activity in several cortical areas related to motor perception, in comparison to controls inexperienced in dance. Gebel et al. (2013) has also recently shown this effect in musicians, finding instrument specific levels of cortical activation between pianists and trumpet players. This clearly demonstrates the effect that expertise can have on neural processing during a task. With regards to feature specific differences in neural processing, Shahin et al. (2008) found a significant enhancement of gamma band activation in musicians for the timbre of their own instrument, using EEG. The current study did not rigorously assess level of expertise in participants past indicating that they were non-expert or novice musical listeners, meaning that the subject group could have been comprised of either a group of more or less musical experience than were investigated in other studies. This may have had an effect on results obtained, as the level of expertise of participants can clearly have an effect on neural activity underlying perceptual processes. In addition to expertise, participants were not rigorously screened for familiarity with the musical piece presented, though on debrief no participant spontaneously reported familiarity with the piece. It has been shown that familiarity arising from multiple presentations of humor reduces ISC (Jääskeläinen et al., 2016). Future research could more carefully control for these factors of expertise and experience.

Obtaining a sufficient sample size to produce reliable results and satisfactory statistical power is a common challenge in fMRI group analysis. Whilst this study made use of 18 participants, in general a sample size of 20–30 is recommended for reliable results in ISC analysis using fMRI (Pajula and Tohka, 2016). Thus, although just outside the margins, the smaller sample size used in this study may affect generalizability and reproducibility of the results found.

## Conclusion

We have explored the ISC of brain activity in a group of observers when watching and listening to ensemble play of a string quartet, and related these findings to the correlation of brain activity to audio and visual features of the performance. Results showed ISC in auditory and visual brain areas that could be anticipated from sensory processing. ISC was also found in parietal cortex, which has been attributed to tracking a signal over time. Finally, cortical and subcortical motor areas that have been related to perceiving rhythmic structure were also implicated. In particular, the left putamen was found both in ISC maps as well as related to the auditory feature of loudness and the visual feature of speed, suggesting that audiovisual interactions of the performance were driving this putamen activity. One possible explanation of the audiovisual interaction comes from the fact that the physical properties of speed and loudness were correlated to each other. However, for the case of movement similarity there was no correlation of this visual feature to an auditory feature. The visual property of movement similarity was related to brain activity in auditory cortex (BA22), with decreasing movement similarity among players leading to higher brain activity. Suggesting that one possible source of audiovisual interaction might be guided by the holistic property of movement similarity, which forms a salient visual feature to facilitate auditory processing (Klucharev et al., 2003; Van Wassenhove et al., 2005; Jessen and Kotz, 2011). These results raise exciting possibilities for future studies that leverage the capability of a relatively short (116 s) scan to reliably localize brain areas related to the processing of naturalistic performance.

One promising avenue for such future research would be the exploration of the ways in which musical structure may lead to convergence and divergence in musicians’ actions and concomitant neural activity. Passages that are largely homophonic could be contrasted with passages of varying degrees of polyphonic complexity as well as interstitial passages that are texturally preparatory or transitional. This should help shed light on neural activity underpinning hierarchically-organized joint action involving formation and dissolution of temporary “coalitions” of musical agents.

## Data availability statement

The raw data supporting the conclusions of this article will be made available by the authors, without undue reservation.

## Ethics statement

The studies involving human participants were reviewed and approved by Ethics Committee of the College of Science and Engineering, University of Glasgow. The patients/participants provided their written informed consent to participate in this study.

## Author contributions

DG, AC, and FP originally conceived the study. AL, DN, DG, BG, and FP performed the data analysis. DG and AC originally obtained the string quartet data who generously provided them for use in this study. BG provided methods for the physical analysis of the auditory signal. IC provided expertise for interpretation of the results in a musical context. AL, DN, and DG contributed to writing the first draft. FP supervised the project. All authors contributed to editing and revising the manuscript.
