# Supplementary figures and images for: A functional magnetic resonance imaging examination of audiovisual observation of a point-light string quartet using intersubject correlation and physical feature analysis
Source: Front Neurosci. 2022 Sep 6;16:921489. doi: 10.3389/fnins.2022.921489 (PMC9486104; doi:10.3389/fnins.2022.921489)

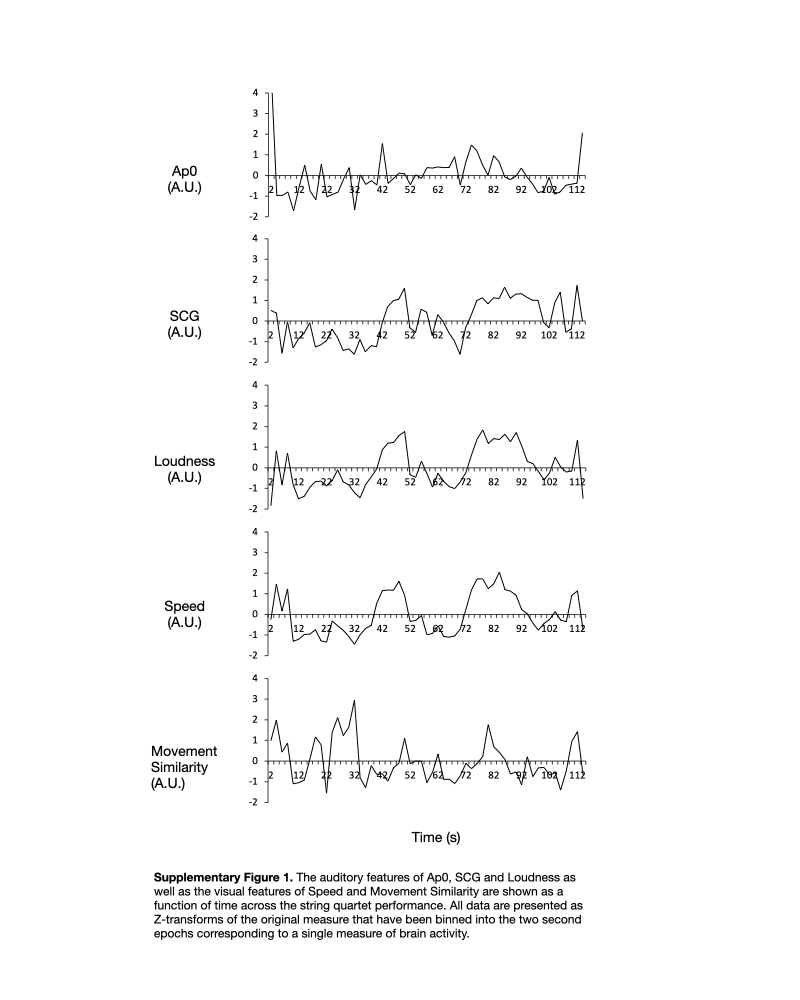

Supplement: Supplementary file 1 [file Image_1.TIF]
